# Familial colonoscopic screening: how do French general practitioners deal with patients and their high-risk relatives. A qualitative study

**DOI:** 10.1080/13814788.2022.2089353

**Published:** 2022-07-07

**Authors:** Isabelle Ingrand, Nicolas Palierne, Pauline Sarrazin, Yvan Desbordes, Clara Blanchard, Pierre Ingrand

**Affiliations:** a INSERM CIC 1402, University Hospital of Poitiers, University of Poitiers, Poitiers, France; b GRESCO (EA 3815), University Hospital of Poitiers, University of Poitiers, Poitiers, France; c Department of General Medicine, University of Poitiers, Poitiers, France

**Keywords:** General practice, prevention, family screening, colorectal cancer, qualitative design

## Abstract

**Background:**

Screening of colorectal cancer (CRC) can reduce incidence and mortality. First-degree relatives (FDRs) of patients with CRC or advanced adenoma before the age of 65 (index patients) are at increased risk of CRC; however, the guidelines for screening of FDRs by colonoscopy are poorly followed.

**Objectives:**

The present study, conducted in the context of the COLOR3 interventional study project, aimed to explore the positioning of general practitioners (GPs) in familial CRC screening in France.

**Methods:**

From February 2020 to April 2021, 35 semi-structured interviews with GPs of index patients and/or their FDRs were conducted by telephone. The full-data transcribed corpus was subjected to horizontal thematic analysis.

**Results:**

Knowledge and compliance with the guidelines vary greatly between GPs. Although initiating the diagnostic process, GPs do not consider themselves as actors in the flow of information concerning familial risk. Their accompaniment of index patients in this role varies. GPs should overcome barriers to implementing colonoscopic screening for FDRs. They underline the importance of exploring family history, but they lack the time and doubt the reliability of the information given by FDRs.

**Conclusion:**

Challenges include circumventing gaps in knowledge, adherence to guidelines and improving family history updates. The GPs interviewed suggested personalised guidelines in specialists' reports to initiate information campaigns raising awareness of familial risk, and to enhance coordination between organised screening and familial screening.

KEY MESSAGES

- General practitioners are central in improving familial colonoscopic screening for high-risk individuals.
- Attention should be devoted to collecting and updating family history.
- General practitioners interviewed suggest incorporating personalised guidelines into reports, campaigns to raise awareness of family risks, and improvement of the articulation with organised screening.

## Introduction

Colorectal cancer (CRC) is a leading cause of cancer burden worldwide. With nearly 45,000 new cases and 18,000 deaths per year, CRC is the third commonest cancer and the second deadliest in France, one of the European countries with the highest incidence of CRC for both genders [1]. Since 1990 the standardised mortality rate has progressively decreased in both males and females, due to earlier diagnosis and better treatment. Five-year survival is 90% if CRC is diagnosed early (tumour confined to the intestinal wall) [2].

Three levels of CRC risk determine differentiated screening and surveillance strategies (Table 1) [3]. The national organised screening (OS) programme, using faecal occult blood testing by immunoassay, targets the first level: average-risk individuals aged 50–74 years without apparent CRC symptoms. The second level is individuals belonging to a family with polyposis or Lynch syndrome (genetic CRC) with a very high risk and requiring specialised oncogenetic management. The third level is two distinct populations considered high-risk and colonoscopic screening is recommended. The first is defined by a personal history of CRC, adenoma or chronic inflammatory bowel disease. The second, the object of the present study, is defined by a family history of CRC or adenoma (familial CRC), occurring in a first-degree relative (FDR) before 65 years, or two or more instances of family history occurring among FDRs, irrespective of age at diagnosis. In the French clinical guidelines, colonoscopic screening of these FDRs is recommended from age 45 years, or 10 years before the age of diagnosis of the index patient, whichever comes first [3]. Access to this familial screening, however, remains low [4]. In the Poitou-Charentes region, starting point of this study, 25% of invasive cancers, and 39% *in situ* CRC and adenomas occur before age 65 years [5,6]. These data suggest that any general practitioner (GP) has at least one index patient and several relatives qualifying for family screening from this group. GPs are central in counselling and motivating patients to undergo colonoscopic screening [7,8]. However, very few studies have focused on the specific role of GPs in familial CRC screening [3], and none on the specific role of GPs towards index patients. In contrast, many studies have been published on GP involvement in organised screening [9–13], and interventions to improve adherence of FDRs to screening [14,15].

**Table 1.** CRC levels of risk and corresponding screening strategies.

	Average risk	Increased risk	Very high risk
Targeted population	General population 50–74 years old Symptom-free	Personal history of chronic inflammatory bowel disease Crohn’s disease Ulcerative colitis History of adenoma or CRC Personal Familial: CRC or adenoma occurring in a first-degree relative before the age of 65, or two or more instances of family history occurring in FDRs, irrespective of age of diagnosis	Inherited predisposition Familial adenomatous polyposis (FAP) Hereditary non-polyposis colorectal cancer (Lynch syndrome)
Screening strategy	Organised screening Faecal occult blood test (every 2 years)	Family individual screening Gastroenterology consultation and follow-up Colonoscopy/chromoendoscopy^a^ from age 45, or 10 years before the age of diagnosis of the index patient, whichever comes first	Individual screening Oncogenetics consultation (search for specific mutation) Gastroenterology consultation Chromoendoscopy^a^

Reproduced from HAS 2017 [5].

^a^Chromoendoscopy is an examination complementary to colonoscopy that consists in marking certain areas of the digestive tract with different dyes, using a spray catheter passed through the operating channel of the endoscope.

This qualitative study aimed to analyse how GPs view their role towards index patients and their high-risk relatives qualifying for indicated familial CRC screening.

## Methods

### Setting

Study data were collected in an interventional study, COLOR3 (ClinicalTrials.gov NCT03620877), aiming to increase participation in colonoscopic screening of FDRs by supporting the coordinated transmission of information from the index patient's GP to the FDR's GP [6]. The GPs solicited were the attending practitioners of index patients treated in Poitou-Charentes and/or their FDRs, residing anywhere in France, who agreed to participate. Sampling was designed to obtain a representative sample of GPs involved in CRC family screening. Gastroenterologists initiated the sampling process for any newly-diagnosed eligible CRC or adenoma. A subsample of 61 GPs was randomly selected from 130 GPs, who were designated by index patients and relatives. Among these 61 GPs, 35 agreed to participate and were interviewed. The resulting sample comprised 18 men and 17 women, aged 35–74 years, and various places of practice (Table 2). GPs who refused to participate mentioned lack of time and involvement in prevention, while others were retired or close to retirement. The reasonably short interviews lasted 10–44 min (20′ on average).

**Table 2.** Characteristics of general practitioners.

Anonymity^a^	Gender	Age	Urban/periurban/rural area (region)
GP1	Female	[60–75]	Periurban (Nouvelle-Aquitaine)
GP6	Male	[30–40]	Urban (Nouvelle-Aquitaine)
GP6-2	Female	[40–50]	Periurban (Nouvelle-Aquitaine)
GP7-1	Female	[40–50]	Periurban (Nouvelle-Aquitaine)
GP8	Male	[50–60]	Rural (Nouvelle-Aquitaine)
GP8-2	Female	[30–40]	Rural (Nouvelle-Aquitaine)
GP10	Male	[40–50]	Rural (Nouvelle-Aquitaine)
GP14	Male	[50–60]	Urban (Nouvelle-Aquitaine)
GP15-1	Female	[40–50]	Periurban (Nouvelle-Aquitaine)
GP15-3	Female	[40–50]	Periurban (Pays de la Loire)
GP16	Male	[60–75]	Urban (Nouvelle-Aquitaine)
GP18-2	Male	[50–60]	Periurban (Nouvelle-Aquitaine)
GP18-3	Male	[50–60]	Periurban (Nouvelle-Aquitaine)
GP19	Female	[40–50]	Periurban (Nouvelle-Aquitaine)
GP20-1	Male	[40–50]	Rural (Occitanie)
GP20-2	Female	[40–50]	Urban (Nouvelle-Aquitaine)
GP23	Female	[50–60]	Rural (Nouvelle-Aquitaine)
GP25	Male	[30–40]	Periurban (Nouvelle-Aquitaine)
GP27	Male	[50–60]	Periurban (Nouvelle-Aquitaine)
GP29-1	Male	[50–60]	Periurban (Nouvelle-Aquitaine)
GP30	Male	[50–60]	Rural (Nouvelle-Aquitaine)
GP-30F	Female	[30–40]	Periurban (Nouvelle-Aquitaine)
GP32-3	Female	[40–50]	Rural (Nouvelle-Aquitaine)
GP44-3	Male	[50–60]	Urban (Occitanie)
GP46	Female	[50–60]	Periurban (Nouvelle-Aquitaine)
GP49	Female	[50–60]	Urban (Nouvelle-Aquitaine)
GP51	Female	[50–60]	Periurban (Nouvelle-Aquitaine)
GP55	Female	[60–75]	Urban (Nouvelle-Aquitaine)
GP56-3	Male	[40–50]	Periurban (Nouvelle-Aquitaine)
GP58	Male	[50–60]	Periurban (Nouvelle-Aquitaine)
GP59	Male	[60–75]	Urban (Nouvelle-Aquitaine)
GP60	Male	[50–60]	Rural (Nouvelle-Aquitaine)
GP61	Female	[30–40]	Urban (Nouvelle-Aquitaine)
GP67	Female	[30–40]	Rural (Nouvelle-Aquitaine)
GP71	Male	[60–75]	Urban (Nouvelle-Aquitaine)

^a^The GP identification corresponds to the number of the patient included in the COLOR3 study.

An index patient’s doctor is identified by this number alone (GP29). A sibling’s doctor is identified by the index patient’s number followed by the sibling's rank (GP29-1, GP29-2…). The physician of the parent of an index patient is identified by the index patient’s number followed by F (father) or M (mother).

### Data collection

Semi-directed telephone interviews, suited to engage with populations with limited time for research [16,17], were conducted by NP (an experienced health sociologist) from February 2020 to April 2021. The interview guide, designed after a review of the literature, pilot interviews and discussions within the research team, focused on GPs' knowledge of guidelines for familial CRC screening, their involvement and discussion with index patients and/or FDRs about the purpose of family screening, the barriers concerning familial CRC screening and their suggestions for improving the implementation of recommendations (Table 3).

**Table 3.** Opening questions and inquiry rationale for semi-structured interviews.

Themes	Questions
Nature of interactions with the index patient or FDR Chronology of discussions about the discovery of the cancer Knowledge of family history	What was the nature of your discussion with your patient – first name, last name – about his/her colorectal cancer?
Discussion of family risk with the index case Assessment of the chronology of the exchanges initiated by the GP, by the patient Assessment of the transmission of the recommendation by the GP Assessment of the GP's role in family screening Assessment of the patient's understanding of the recommendation Assessment of the limitations of the family screening	Did you discuss this issue of familial risk with your patient? If you did, what was the nature of the discussion? If not, what difficulties did you encounter?
Discussion of family risk with the FDR Assessment of the chronology of the exchanges initiated by the GP, by the patient Assessment of the transmission of the recommendation by the GP Assessment of the GP's role in family screening Assessment of the patient's understanding of the recommendation Assessment of the limitations of the family screening	Did you discuss this issue of familial risk with your patient? If you did, what was the nature of the discussion? If not, what difficulties did you encounter?
Possible FDRs in the same practice as the index patient	Do you have any index patient's FDRs in your practice? Have you spoken with any of them about high risk?
Questioning on family history Questioning of the GP/spontaneous statement of the patient In which consultation settings do you talk to your patients about their family history of cancer?	In which consultation contexts do you talk to your patients about their family history of cancer?
Knowledge of guidelines for CRC family screening	Around what age do you refer relatives for colonoscopy?
Suggestions for improving the implementation of the recommendations	What tools would you find useful to improve your knowledge of your patient’s family history?

### Data analysis

The interviews were audio-recorded and transcribed anonymously. The corpus was analysed thematically [18]. Transcripts were read and initial codes were generated (NP, PS, CB, II) and discussed at research meetings. Theory triangulation and pluridisciplinary interpretation of the data were performed by GPs (PS, CB, ID), sociologists (NP) and public-health professionals (II, PI) [19]. The researchers read the transcripts independently, re-read them together and discussed them until they reached a joint solution. The study was conducted and reported using the Consolidated Criteria for Reporting Qualitative Research (COREQ).

### Ethics

Ethics approval was received from the CPP Northwest I on 27 February 2018 (Ref CPP: N° CPP 005/2018 - N°RCB: 2017-A03445-48). The study protocol was also registered in ClinicalTrials.gov (Identifier: NCT03620877).

## Results

Themes and categories emerging from the analysis are summarised in Table 4. These included GPs' knowledge of the guidelines, their involvement with index patients and relatives, barriers to family CRC screening, and proposals to improve it.

**Table 4.** Themes and categories developed from GPs’ interview data about familial CRC screening.

Themes	Knowledge of guidelines	Involvement with index patients	Involvement with high-risk relatives	Barriers for GPs to envisage family CRC	Proposals for improving the implementation of the guidelines
Categories	Identifying high-risk relatives Age at diagnosis of index patient Age of the first colonoscopy for FDRs Adenomas included in the guidelines alongside cancers	Recalling the guidelines Relaying the recommendation Supporting the patient during the process Replacing the patient if necessary	Investigating the level of risk Questioning about family history Coordination with organised screening Fear of missing early detection	Lack of time or availability Lack of involvement in prevention Lack of personalised guidelines Incomplete family history	Guidelines included in the reports Improved organised screening and awareness campaigns Updating family history A similar organisation to that of oncogenetic consultations

### Knowledge of guidelines

#### Identifying high-risk relatives

The identification of FDRs (parents, siblings, children) indicated by the guidelines was performed by the GPs interviewed. Only one GP reported that she did not know that parents were also high-risk FDRs.

*It's true that I didn't think that when children have problems, we should also look at the parents. (GP30)*

#### Age at diagnosis of index patient and age of the first colonoscopy for FDRs

When quoting the recommendations, most GPs did not mention the age of the index patient at diagnosis, and were unsure at what age FDRs should start colonoscopic follow-up. Several mentioned the age of 35 or 40 years without mentioning the 10-year criterion preceding the age of the index patient at diagnosis.

*I'll be honest. I say, from 35 years old, in fact…, it's more or less what I do! (GP58)*

#### Adenomas included in the guidelines alongside cancers

While GPs fully associated CRC with the screening guidelines, none clearly knew the characteristics of adenomas falling within the scope of the guidelines and some (5/21 GPs of index patients) were unaware that a patient with one or more adenomas was an index patient.

### Involvement with index patients

Although GPs acknowledged their central role in familial CRC prevention for relatives of index patients, they did not feel they were in the best position to say which patients met the criteria to be considered as index patients for their relatives, contrasting specialists (gastroenterologists, digestive surgeons and oncologists), who routinely collect and interpret anatomopathological reports. All interviewed GPs acknowledged their initiating role in the diagnostic process by referring the patient to gastroenterologists, for symptoms or a positive immunological test, and then ‘handing over’. They did not consider themselves as primary actors in identifying index cases, leaving this to specialists but indicated their willingness to be involved in the process. Their involvement could address recalling the guidelines, ensuring information is relayed, supporting the patient during this process, or, more rarely, relaying it themselves to family members with the patient's consent.

#### Recalling the guidelines

For GPs, recalling and explaining the guidelines helps to ensure that the information is properly understood and relayed within the family.

*We explain to them again, when we see them, that it is because there is a risk, so that they in turn pass on the knowledge to their children, to their nephews, so that […] the information is not lost over time. (GP18-3)*

Some GPs expressed reluctance to mention the guidelines when index patients still demanded other, usual care, and waited for the right moment.

*It would be more anxiety-provoking than anything else for the patient. […] It might also make him feel guilty towards his children. […] I prefer to wait for a little until things are quite stable. (GP6)*

However, for some, this backup can be limited by GPS' lack of knowledge of family composition (geographical distance from relatives, substitute physician, recent removal), which tends to reduce discussions with children. Different cognitive biases could also occur.

*He is living with a man […] I'm not sure he has children. (GP27)*

*I'll be honest, I deal more with the medico-social problems [smile] than the purely medical ones. (GP58)*

#### Relaying the recommendations

GPs emphasise the responsibility of index patients in passing through the recommendations. On the one hand, they believe that patients welcome it to protect their relatives.

*In general, they are pleased to have this to do because they seem to think that they will be able to avoid [smile] others in their family having the same worries as themselves. (GP1)*

On the other hand, they see this as a ‘duty’ imposed on their patients, and one GP preferred to relay the information himself to alleviate the burden on the index patient. This is particularly true because attending GPs are no longer the ‘family doctor’, following changes in family configurations and urbanisation. The GPs also mentioned cases of estrangement or family conflicts.

*Sometimes they tell us that they no longer have contact with this or that person in their family, that they don't even know what are their their family’s health problems. […] Some people are angry, they don't talk to each other anymore. (GP1)*

However, some emphasised that they had no feedback on the fate of relatives.

*It's true that afterwards, the steps are taken by the patient himself, eh? […] I haven't had any feedback at all. (GP10)*

GPs mentioned strategies to ensure that the diagnosis was known to relatives without breaching medical confidentiality.

*If, for example, I know someone, I discover their father has had colon cancer when I see that person again, I say: Well, how is your father? If he doesn't tell me anything, I just don't say anything. If he says: “Oh yes, he had an operation, he had colon cancer”, then I say: Well, were you warned that…. you too should be monitored? […] I'm not the one who's going to tell him about his father's cancer. (GP30)*

#### Supporting the patient during this process

Index patients have asked GPs to provide leaflets to discuss the family risk with relatives.

*Well, they ask whether there should be screening for descendants or brothers and sisters. […] They ask for leaflets, for example, so that they can talk about it with their relatives. (GP23)*

#### Replacing the patient if necessary

Finally, a few GPs reported relaying the recommendations directly to relatives. Some patients did not feel they were in the best position.

*People with little education. They don't necessarily understand the disease very well. […] And as a result, they [the index patients] say: 'Well, I prefer you to talk about it. […] Because, maybe they are afraid of being too alarming. (GP67)*

One index patient died early, so a GP and her colleagues took on the responsibility of counselling the relatives (GP67).

### Involvement with high-risk relatives

#### Investigating the level of risk

GPs specified their role with FDRs: investigating and informing on the level of risk, proposing appropriate screening and initiating procedures. For GPs interviewed, different opportunities facilitated this: OS or any opportunity to update family history or the presence of symptoms.

#### Articulation with organised screening

In France, OS offers the opportunity to detect patients at higher risk via a review of the family history.

*When they receive the letter from Docvie [screening structure], I ask them […] if they have a family history, which means that at that point, the test, in quotes, is not useful, and it is better to go directly to the gastroenterologists for the colonoscopy. (GP19)*

OS has helped raise patient awareness of the different levels of risk and the associated guidelines cited in the letters. However, support from the GP to tailor this information is still necessary.

*She [FDR] said to me I don't understand; now they're telling me that I have to do [the colonoscopy] even though the [immunological] test is negative. (GP18-3)*

Awareness determines the participation of patients in OS, and for several GPs, patients attend the tests but with another reason for consultation. The desire of some to have the test without a consultation, and the lack of availability of GPs, could jeopardise this link between OS and family screening. Some GPs state that they prescribe the test in most cases, while others say their secretary deals with it. They point out that they may have missed some at-risk relatives in the OS because they were unaware of the family history.

*The Haemoccult tests may have been performed twice, three times, and then […] a family history can occur in between. The patient didn't necessarily read the leaflet he had received properly! […] That's it, so you must be on the lookout. (GP18-2)*

#### Questioning about family history

The GPs interviewed emphasised their central role in family screening for FDRs using family history.

*I think our role is essential. Provided that we have the possibility of devoting time to it. […] Afterwards, the patient says what he or she wants to say! (GP18-2)*

In addition to OS, the presence of symptoms is a major reason for questioning.

*Whenever you have abdominal pain that seems a bit suspicious and so on, indeed this is always on your mind (GP46)*

However, questioning is conditioned both by the time GPs have and by the quality of the answers given by relatives. To justify their lack of time to pursue the questioning, GPs mentioned the complexity of consultations dealing with multiple demands, added complexity in rural practices, the hospital-centred nature of curative care, and the multitudes of guidelines. They also mentioned ignorance of familial risks and family history among FDRs.

*People often say, well he had something removed, we don’t really know what it was. It’s complicated if you don’t know exactly what it is … what the actual diagnosis is. (GP15-3)*

They encourage patients to ask their relatives for information and make them aware of family risks as soon as the file is created.

*If something happens in your family, well, it's perhaps important to tell me about it too. They're all fine, but maybe one day they'll have a health problem. It may concern you directly. (GP67)*

Due to breast and cervical OS, women are more aware of cancer prevention, especially with female GPs. Several GPs underlined a greater willingness among women to participate in prevention, both for themselves and those around them.

*They are more sensitive to prevention. […] Paying attention maybe to others, too. (GP27)*

#### Fear of missing early detection

GPs report being particularly vigilant towards inaccurate information on family history or poor understanding of family screening guidelines.

*They say, well there were polyps. I know I will never have the pathology results, so I refer in excess rather than by default. (GP67)*

They tend to refer relatives earlier than recommended, which reassures the worried patient and leaves the decision to the gastroenterologist.

*When in doubt, I suggest, and then it is the gastroenterologist who, in the end, makes the decision and starts the screening. [**…**] If the gastroenterologist considers, even if the age is not quite right, it is more prudent [**…**]. It is he who decides. (GP23)*

Despite sometimes over-referring, these GPs remained aware that colonoscopy is not risk-free.

### Barriers for GPs in performing family screening

The first barriers for GPs to screen for familial CRC are most certainly those they themselves mentioned for not participating, namely the lack of time for prevention and primacy of curative care in their practices. The lack of knowledge of the guidelines (in particular age at diagnosis and age for initiating surveillance) led some GPs to consider that their patients, especially the relatives were not concerned by family screening. They also mentioned the absence of age criteria for starting colonoscopy surveillance in the reports sent them by specialists. Poor family history documentation means GPs do not refer their patients to appropriate screening (immunological test, colonoscopy, oncogenetic consultation).

### Proposals for improving the implementation of the guidelines

#### Guidelines included in the reports

In line with cervical cancer screening, GPs suggest that the family risk of CRC should be notified in the pathology and/or colonoscopy reports, with a reminder of the guidelines and personalised management strategies for relatives. More specifically, several GPs would like guidelines on the age at which colonoscopy is recommended for children whose parents are still young at the time of diagnosis, suggesting a lack of knowledge of specific guidelines.

*If the GP is informed, that's good. If the GP is warned, that's good. […] So that's rather positive because it's true that sometimes they don't have the information, or they don't really know […] (GP15-1)*

All GPs of FDRs approved the letter sent in the COLOR3 study, mentioning the exact nature of the family history and age at which the event occurred.

#### Improved organised screening and awareness campaigns

GPs interviewed felt that improving OS would improve family screening. Some GPs considered that a prevention nurse could assist in updating the family history before prescribing the risk-level-appropriate test. Some suggested large-scale media campaigns, like those on antibiotics, to encourage people to talk about their family history.

*It would be interesting if there was a public campaign… wider, […] Because I've had people who were completely unaware. (GP29-1)*

#### A similar organisation to that of oncogenetic consultations

GP10 mentioned the multidisciplinary work on oncogenetic screening (breast and CRC) and suggested instating follow-up of high-risk relatives on the same model, including computerised tools. On receipt of a genetic consultation report, the GP would be informed that his patient has been diagnosed with a genetic predisposition to cancer and that the information should be relayed to his relatives [20].

## Discussion

### Main findings

Drawing on interviews with French GPs, this study is the first to examine their positions not only towards high-risk relatives requiring family screening but also towards index patients diagnosed with CRC or adenoma before 65 years.

GPs play an initiating role in the diagnostic process and a role in specialist referrals. Interviewed GPs said they were not very involved in supporting index patients, first because they tended to leave this to specialists, and secondly, they feel that the index patient should relay the recommendations to their FDRs. However, some explain the recommendations to ensure the information is understood correctly and thus better relayed to the family. A few counselled FDRs directly. With FDRs, their role is to inform and investigate the level of risk, suggest appropriate screening and initiate action. All GPs interviewed emphasised their central role in CRC family screening in exploring family history but stressed the constraint of the time they can devote to it and the variable quality of answers given by relatives. Indeed, knowledge of a family history of CRC or adenoma leads GPs to refer patients for colonoscopy rather than immunological testing. Uncertainty around the exact details of the family history is a considerable difficulty in family screening and could contribute to precautionary referrals to gastroenterologists. However, GPs interviewed stressed that the benefit-risk balance of a colonoscopy was the central concern.

### Strengths and limitations

These GP perspectives offer valuable insights into an area of scant research. GPs with diverse experiences and practising across France were interviewed. They made proposals for better implementation of the guidelines.

GPs were informed before the interview that the study focused on familial CRC screening, which could have influenced their responses or decision to participate. GPs who never discussed risk with their patients or did not propose colonoscopy screening possibly refused to participate. Responses concerning their knowledge of the recommendations may have been biased. Some claimed to know and apply the guidelines, without describing their actual practice. Others gave incorrect, elusive or allusive answers. These biased answers reflect the difference between what people say and what they do but probably do not challenge the findings on barriers to colonoscopy screening and suggestions for improvement.

### Comparison with existing literature

Poorly understood guidelines [21], and a growing body of literature can confuse GPs [22,23]. Gastroenterologists, who diagnose CRC and adenomas, are ideally positioned to inform patients about screening guidelines [7]. Some GPs said they relayed the guidelines but none ensured that index patients understood the value of colonoscopy for their FDRs [24,25]. Although some GPs felt that index patients themselves were the best placed to advise their FDRs on the benefits of screening [21], leaving the task of family risk prevention to patients and families could exacerbate social health inequalities (exposure to risk, willingness for prevention and care, understanding medical issues). None of the interviewed GPs mentioned the presence of information relays (nurses) under their control who could ensure that patients understand the diagnosis and guidelines and possibly relay the information to relatives [26]. None suggested passing information directly from the index case doctor to the relative's doctor. Current legislation on patient confidentiality prevents doctors from communicating directly with their patients' relatives, but with their patient’s agreement they can communicate with their FDRs’ doctors [6].

The updating of family histories is constrained by the time GPs can spare, and the information given by relatives [7,21]. Thus, despite the undisputed fact that physician endorsement is a key factor in participation in family screening [27], this study confirms that the GPs interviewed did not regularly ask their patients about their family history. Systematic and cost-effective procedures are required to facilitate family history updating for risk assessment and to deliver screening advice within the primary health care setting [28,29]. Being aware of a family history of CRC is related to greater adherence to CRC OS [30]; updating family history is also an essential step in the process of CRC OS to correctly evaluate the risk level and propose an adequate screening strategy [30].

The GPs are best placed to improve awareness of CRC susceptibility and remove barriers to screening among FDRs. However, in addition to patient and system-level barriers, there are GP barriers. Challenges include poor knowledge of the guidelines and lack of adherence to them, time constraints, inaccurate and incomplete family history data [31].

### Implications for research and practice

Family screening relies on accurate identification of index cases and knowledge of the guidelines for their high-risk FDRs. According to the GPs interviewed, personalised guidelines could be sent to the GP with colonoscopy, surgery, and pathological results, since reports are a reliable resource easily used in primary care settings.

OS for CRC among medium-risk 50–74-year-olds offers GPs an opportunity to ask their patients about their family history and direct them to the appropriate screening strategy. According to the GPs interviewed, coordination between OS and familial screening should be improved and public information campaigns would make families aware of the importance of family history and its utility in terms of risk assessment.

Assistance in relaying information, such as specially trained nurses, could ensure that patients understand the diagnosis and guidelines and relay the information to relatives.

Relevant, effective public health interventions should focus on the coordinated transmission of medical information from the index case’s GP to the relatives’ GPs.

## Conclusion

GPs interviewed recognised their central role in improving adherence to familial CRC screening guidelines towards high-risk relatives for whom it is recommended. GPs also endorse the responsibility of recalling and explaining the guidelines toward index patients diagnosed with CRC or adenoma before 65 years of age. Challenges include filling gaps in knowledge, adherence to the guidelines and improving family history updates. The GPs suggested integrating personalised guidelines into specialists' reports, to efficiently articulate organised family screening, and initiate information campaigns to raise awareness of family risks.
